# Diagnostic and screening utility of various test methods for malaria – a comparative study of malarial parasites in blood smear, quantitative buffy coat and detection of malarial antigen by immunochromatography and ELISA

**DOI:** 10.1186/1471-2334-12-S1-P81

**Published:** 2012-05-04

**Authors:** A Ashwin, Febe R Suman, Vinod K Panicker, S Alexander, R Krishnamoorthy

**Affiliations:** 1 Department of Transfusion Medicine, Sri Ramachandra University, Chennai, India

## Background

Malaria causes about 250 million cases of fever and 1 million deaths annually. It is one of the causes of poverty and a major hindrance to economic development. Malaria is caused by protozoa of the genus plasmodium. Early accurate diagnosis is essential to start treatment within 24 hours and to avoid empirical therapy with toxic drugs. This study is aimed at finding out the best diagnostic and screening method for malaria.

## Methods

Blood from 200 clinically suspected malaria patients were subjected to peripheral blood smear examination, quantitative buffy coat, immunochromatography and enzyme linked immunosorbent assay. The sensitivity and specificity were analyzed. The duration, equipment, expertise, electricity and economy needed were considered.

## Results

Peripheral blood smear is highly reliable, species specific, requires expertise and is time consuming. Quantitative buffy coat is a reliable rapid test, but needs economy and experts. Immunochromatography is a rapid, economical, low sensitivity method. Enzyme linked immunosorbant assay is a economical, time consuming, low sensitivity method.

## Conclusion

Peripheral blood smear remains the gold standard technique while quantitative buffy coat is the most suitable rapid test in well equipped laboratories and blood banks with heavy work load. Immunochromatography and enzyme linked immunosorbent assay are suitable for population screening in endemic areas and to monitor anti-malarial treatment.

